# The effects of saturated and unsaturated fatty acids on MASLD: a Mendelian randomization analysis and in vivo experiment

**DOI:** 10.1007/s00394-024-03560-2

**Published:** 2024-12-24

**Authors:** Fengming Xu, Mohamed Albadry, Annika Döding, Xinpei Chen, Olaf Dirsch, Ulrike Schulze-Späte, Uta Dahmen

**Affiliations:** 1 Experimental Transplantation Surgery, Department of General, Visceral and Vascular Surgery, Jena University Hospital, 07747 Jena, Germany; 2 Else Kröner Graduate School for Medical Students “JSAM”, Jena University Hospital, 07747 Jena, Germany; 3 Department of Infectious Diseases, The First Affiliated Hospital of Zhejiang Chinese Medical University, Hangzhou, 310006 China; 4 Department of Pathology, Faculty of Veterinary Medicine, Menoufia University, Shebin El Kom , 6131567 Egypt; 5 Section of Geriodontics, Department of Conservative Dentistry and Periodontics, Jena University Hospital, 07743 Jena, Germany; 6 Institute for Pathology, BG Klinikum Berlin, 12683 Berlin, Germany

**Keywords:** Unsaturated fatty acid, MASLD, Hepatic steatosis, Risk factor, Mendelian randomization

## Abstract

**Background:**

Excessive intake of fatty acids is a key factor contributing to metabolic dysfunction-associated steatotic liver disease (MASLD). However, the effects of saturated fatty acids (SFA) and unsaturated fatty acids (UFA) on the development of MASLD are uncertain. Therefore, we conducted two-sample Mendelian randomization studies and animal experiments to explore the effects of SFA, monounsaturated fatty acids (MUFA) and polyunsaturated fatty acids (PUFA) on the risk of developing MASLD.

**Methods:**

The genetic summary data of exposures and outcome were retrieved from genome-wide association studies (GWASs) and used for five Mendelian randomization methods. A comprehensive sensitivity analysis was performed to verify the robustness of the results. Mice were subjected to different diets followed by assessment of severity of steatosis based on a histological score and determination of hepatic triglyceride levels to investigate the relationships between SFA, MUFA, PUFA and MASLD.

**Results:**

The Mendelian randomization results showed that MUFA (odds ratio: 1.441, 95% confidence interval: 1.078–1.927, *P =* 0.014) was causally associated with the incidence of MASLD. SFA and PUFA were not causally associated with the incidence of MASLD. Sensitivity analysis did not identify any significant bias in the results. The animal experiment results showed that a MUFA-enriched diet significantly contributed to the development of hepatic steatosis (*P <* 0.001).

**Conclusion:**

SFA and PUFA did not have a significant causal effect on MASLD, but MUFA intake is a risk factor for MASLD. A MUFA-enriched diet increased the incidence of macrovesicular steatosis and the hepatic triglyceride levels. Therefore, replacing MUFA intake with a moderate intake of PUFA might help reduce the risk of MASLD.

**Graphical Abstract:**

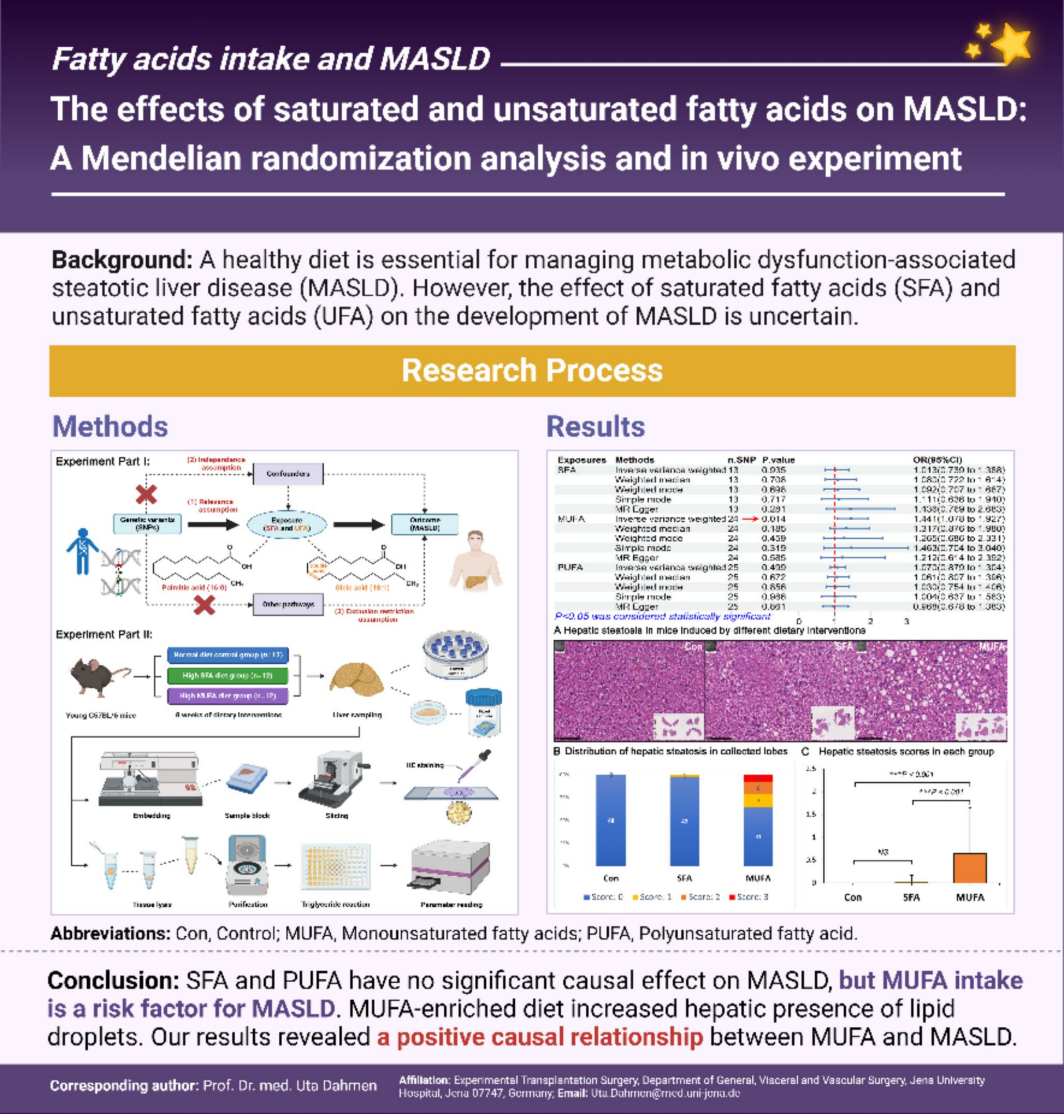

**Supplementary Information:**

The online version contains supplementary material available at 10.1007/s00394-024-03560-2.

## Introduction

Metabolic dysfunction-associated steatotic liver disease (MASLD), formerly known as Non-alcoholic fatty liver disease (NAFLD), has become the most common chronic liver disease worldwide, placing an unprecedented burden on the healthcare system [1, 2]. A systematic review study showed that the global prevalence of MASLD in the adult population was approximately 32.4%, which is much higher than previous estimates and continues to increase at an alarming rate [3]. In developed nations, MASLD has become a primary contributor to cirrhosis and liver cancer [4–6]. As the number of patients with MASLD continues to increase, it is becoming increasingly important to identify and reduce the risk associated with the development of MASLD.

Dietary habits play a crucial role in the development of MASLD. MASLD is characterized by the presence of more than 5% lipid-laden hepatocytes without a competing cause of hepatic steatosis [7]. Excessive intake of fatty acids is known to be one of the pivotal risk factors for MASLD [8]. Saturated fatty acids (SFA) and unsaturated fatty acids (UFA) are the two main types of fatty acids, where UFA can be further classified into monounsaturated (MUFA) and polyunsaturated fatty acids (PUFA) based on their structure. SFA is often considered unhealthy as it is believed to be associated with adverse effects such as insulin resistance, increase of low-density lipoprotein (LDL) cholesterol and an over-all pro-inflammatory condition [9, 10]. In contrast, consumption of UFA is usually recommended due to its presumable antioxidant and anti-inflammatory effects [11]. However, the above view has been challenged over the past decade. Accumulating evidence has shown that SFA does not significantly exacerbate the risk of coronary heart disease (CHD). In contrast, the use of UFA as a substitute for SFA did indeed increase the risk of CHD [12, 13]. Similar findings have been reported in studies of MASLD [14, 15].

The liver is a pivotal organ in fatty acid metabolism. Fatty acids accumulate in the liver through uptake from plasma by hepatocytes and by de novo biosynthesis. Fatty acids are eliminated via oxidation within hepatocytes or by secretion into plasma via triglyceride-rich LDLs. Under normal conditions, the liver stores only small amounts of fatty acids in the form of triglycerides. In cases of overnutrition, fatty acid metabolism in the liver is altered, usually resulting in the buildup of triglycerides in hepatocytes and triggering the clinical condition known as MASLD [16, 17]. However, the role of SFA and UFA in the development of MASLD remains uncertain.

For example, palmitic acid (PA) is the most common SFA found in animals. It is often recognized as having adverse effects on chronic diseases in adults. For instance, high intake of PA increases the risk of cardiovascular disease (CVD) [18–20]. In contrast, consumption of oleic acid (OA), a monounsaturated omega-9 fatty acid, is considered to be more beneficial to health than is consumption of PA, as it is associated with a reduced incidence of CVD [21]. However, Eynaudi et al. reported that the administration of 200 µM OA to HepG2 cells promoted more and greater lipid accumulation than did the administration of 200 µM PA [14]. Similar results were observed by Ricchi et al. They reported that 0.66 mM OA induced substantially greater triglyceride accumulation than 0.66 mM PA in HepG2, HuH-7 and WRL-68 cells [15]. In contrast, Zeng et al. showed that partial replacement of a high-fat diet (high in SFA) with olive oil (high in MUFA) substantially improved hepatic steatosis in Sprague‒Dawley rats [22]. Therefore, the effects of SFA and UFA on MASLD still need to be clarified.

Traditional observational studies are not sufficiently persuasive since they are susceptible to a large number of confounding factors and reverse causality [23]. Currently, Mendelian randomization (MR) has emerged as a powerful method for identifying causal relationships between risk factors and diseases via genetic variants (single nucleotide polymorphisms, SNPs) analysis [24, 25]. MR uses genetic variants randomly assigned at conception as instrumental variables (IVs) to estimate the causal effect of exposure on outcome and can lessen bias due to confounders or reverse causation [26].

Furthermore, the diagnosis of MASLD requires evidence of hepatic steatosis, which is dependent on imaging techniques used in clinical practice. In addition, liver biopsy remains the gold standard for evaluating and diagnosing MASLD [27]. Therefore, we performed animal experiments and collected liver specimens to determine the severity of steatosis developing in response to diets differing in SFA and in UFA. Using this combined approach, we want to clarify the specific effects of SFA and UFA on the development of MASLD.

An in-depth understanding of the effects of SFA and UFA on the risk of MASLD could help to develop more precise and effective prevention and treatment strategies. Such strategies could contribute to slow down the growing global prevalence of MASLD and reduce the incidence of MASLD-related malignant liver diseases. To achieve these goals, we conducted two-sample Mendelian randomization (TSMR) studies as well as animal experiments to further explore the effects of SFA, MUFA and PUFA on the incidence of MASLD.

## Materials and methods

### Study design

In this study, we first carefully explored the causal relationships between SFA, MUFA, PUFA and MASLD through TSMR studies. Subsequently, we further validated the findings through corresponding animal experiments and ultimately drew conclusion.

**Fig. 1:**
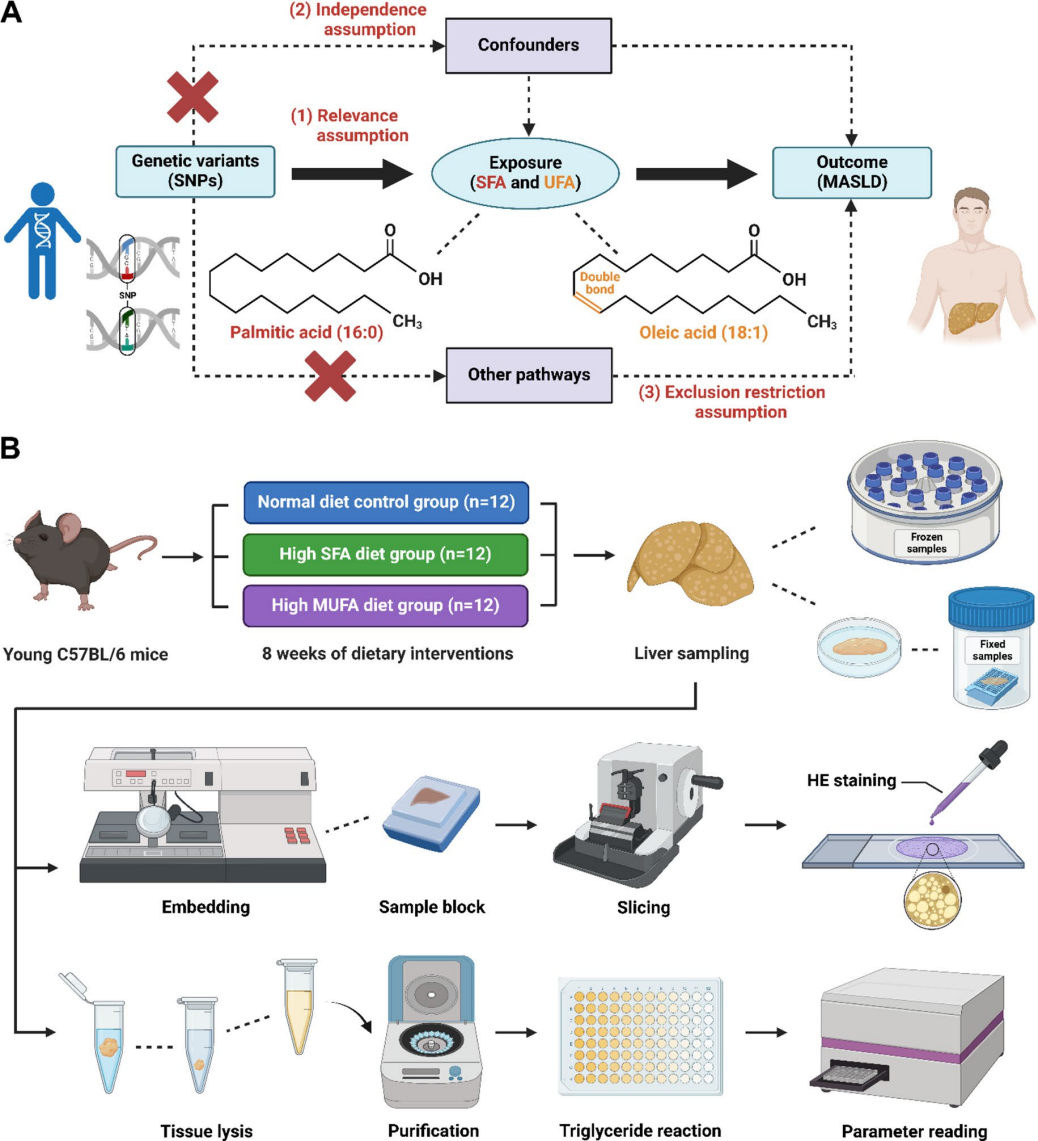
Work flow for investigating the relationships between SFA, MUFA, PUFA and MASLD via TSMR studies and animal experiments. (**A**) The causal relationships between SFA, UFA (MUFA and PUFA) and MASLD were explored via the TSMR analysis. The concepts and three core assumptions of the TSMR analysis are shown. (**B**) The relationships between control diet, SFA-enriched diet, MUFA-enriched diet and hepatic lipid levels were further validated in animal experiments. We created the figure with “BioRender.com.”

In our TSMR experiments, SFA, MUFA and PUFA were used as exposure factors, and MASLD was used as an outcome measure. Three core assumptions need to be met to conduct TSMR analysis: (1) the selected SNPs should be significantly associated with exposure (SFA, MUFA and PUFA); (2) the selected SNPs should be independent of confounders; and (3) the selected SNPs should be associated with the outcome (MASLD) only via exposure (Fig. 1A).

In our animal experiments, we randomly divided the C57BL/6 mice into 3 groups: (1) the normal diet control group (*n* = 12); (2) the high SFA diet group (diets high in PA, *n* = 12); and (3) the high MUFA diet group (diets high in OA, *n* = 12). After 8 weeks of dietary intervention, the mice were sacrificed, and liver samples were collected for histological assessment of steatosis severity and for determination of hepatic lipid levels (Fig. 1B).

### Animals

Male C57BL/6 mice weighing 24–31 g, aged about 13 weeks, were purchased from Janvier (CS 4105 Le Genest St Isle, 53941 Saint Berthevin Cedex, France) and were maintained in a 14/10 h light-dark cycle in groups of three per IVC cage supplemented with standardized enrichment according to ARRIVE guidelines. The animals were included in the trial after an acclimatization period of at least one week and received the respective dietary intervention over a period of 8 weeks. Animals were assigned blind codes and used throughout the experiment. Animals received either high-SFA (containing 9.3% palm oil and 9.3% palmitate ethyl ester) or high-MUFA (containing 18.6% HO sunflower oil) diets compared to animals fed a normal chow diet (containing 3.1% HO sunflower oil, Ssniff, GmbH). Food and water were provided ad libitum. The average daily calories from fat per mouse in the normal diet group were approximately 1.151–2.302 kcal, and the average daily calories from fat per mouse in the SFA and MUFA groups were about 5.245–10.490 kcal, depending on the individual food consumption. To minimize stress, scoring, weighing and food replacement were performed at the same time wherever possible. After 8 weeks of dietary intervention, the mice were sacrificed, and liver samples were collected for analysis of hepatic lipids.

### Ethics statement

In our TSMR experiment, we used summary data from a publicly available database (OpenGWAS) that had obtained participant consent and ethical approval. The data that support the findings of this study are openly available in the OpenGWAS database (https://gwas.mrcieu.ac.uk/), reference number (SFA: ebi-a-GCST90092980; MUFA: ebi-a-GCST90092928, PUFA: ebi-a-GCST90092939 and MASLD: ebi-a-GCST90054782).

In our animal experiment, all procedures were performed in accordance with the German Law on the Protection of Animals/European Communities Council Directive (86/609/EEC) and approved by the Thuringia State Office for Food Safety and Consumer Protection (Approval number: UKJ-17/036).

### Data sources

SNPs for SFA were extracted from a genome-wide association study (GWAS) dataset (ID: ebi-a-GCST90092980) that included 115,006 samples of European ancestry; SNPs for MUFA were extracted from a GWAS dataset (ID: ebi-a-GCST90092928) that included 115,006 samples of European ancestry; SNPs for PUFA were extracted from a GWAS dataset (ID: ebi-a-GCST90092939) that included 115,006 samples of European ancestry [28]; and summary statistical data for MASLD (sample size: 377,998) were obtained from a GWAS dataset (ID: ebi-a-GCST90054782) of European ancestry [29]. The specific summary information is shown in Table 1.

**Table 1:** Characteristics of the data used in the mendelian randomization study

Exposures /Outcome	GWAS ID	Ethnicity	Year	Sample size	Number of SNP	PMID
SFA	ebi-a-GCST90092980	European	2022	115,006	11,590,399	35213538
MUFA	ebi-a-GCST90092928	European	2022	115,006	11,590,399	35213538
PUFA	ebi-a-GCST90092939	European	2022	115,006	11,590,399	35213538
MASLD	ebi-a-GCST90054782	European	2021	377,998	9,097,254	34535985

### IVs selection criteria

**Fig. 2:**
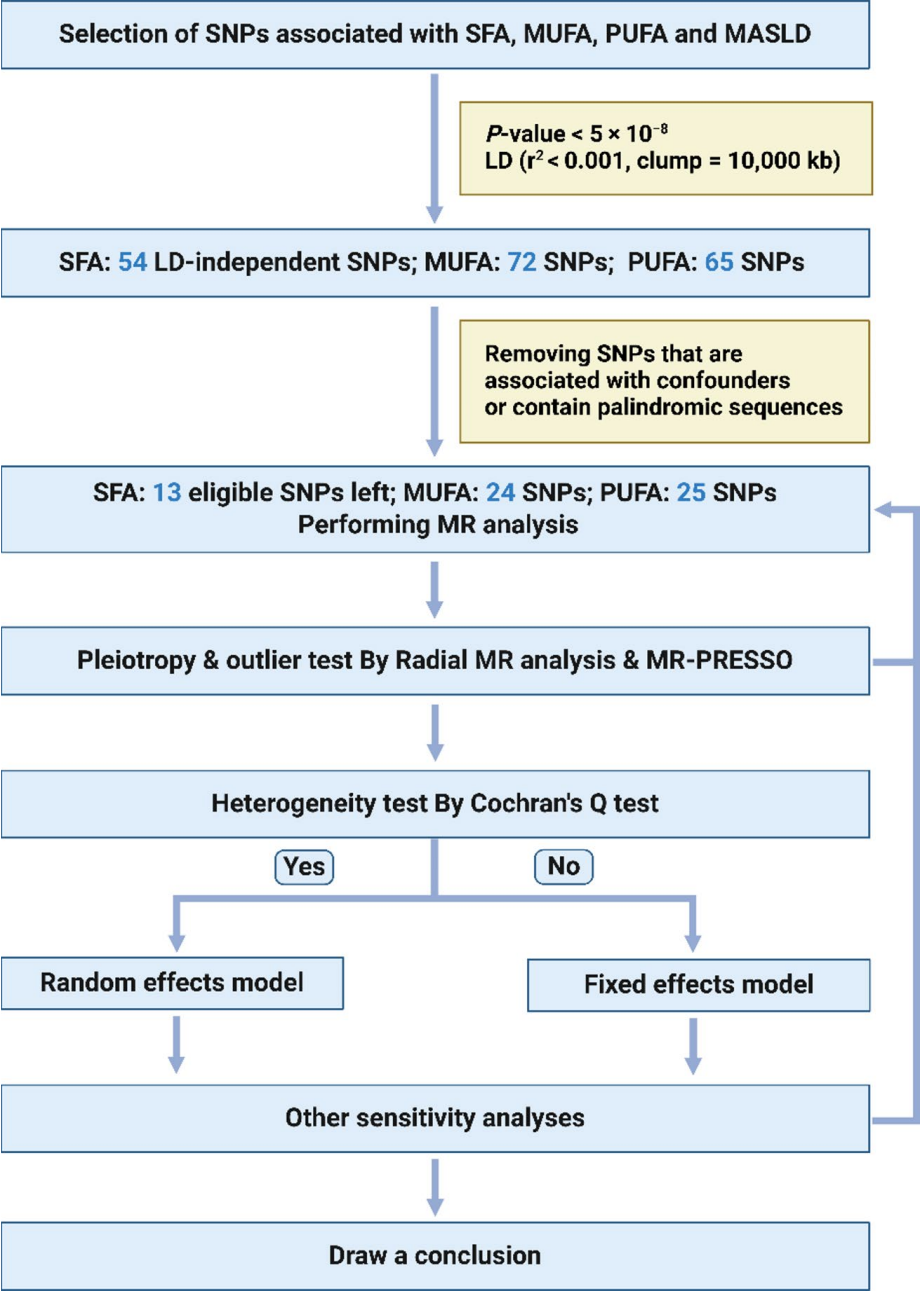
Flow diagram of the TSMR study. Abbreviations: MR, Mendelian randomization; LD, linkage disequilibrium; SNP, single nucleotide polymorphism. We created the figure with “BioRender.com.”

We selected significant and independent SNPs for exposure factors (SFA, MUFA and PUFA) as IVs based on the following criteria:

(1) SNPs that reached a genome-wide association significance level of *P <* 5 × 10^− 8^;

(2) SNPs were independent, and the threshold of linkage disequilibrium (LD) was r^2^ < 0.001 and clumping window = 10,000 kb (Fig. 2);

(3) SNPs with F-statistics < 10 were excluded to avoid weak instrumental bias, and the calculation formula was F = ((*N*-*K*-1)/*K*)*(*R*^2/(1-*R*^2)). In the above formula, *R*^*2*^ represents the cumulative explained variance of the selected SNPs during exposure. *N* is the sample size of the exposure database, and *K* is the number of SNPs included in the analysis. F-statistics > 10 indicates a low likelihood of weak instrument bias;

(4) SNPs in the presence of palindromic sequences were excluded;

(5) SNPs associated (*P* < 1 × 10^− 5^) with known confounders (e.g. Hypercholesteremia, Diabetes, Hypertension, Obesity, Hyperthyroidism, Hypothyroidism, Myxoedema, Disorders of parathyroid gland, Inflammatory bowel disease, High-density lipoprotein cholesterol change with statins, Taking cholesterol-lowering medication) were removed [30]. SNP-related phenotypes were searched via a database of human genotype-phenotype associations (http://www.phenoscanner.medschl.cam.ac.uk/).

Following the rigorous screening criteria mentioned above, 13 SNPs were used as IVs to investigate the causal relationship between SFA and MASLD (Table S1); 24 SNPs were employed as IVs to study the causal relationship between MUFA and MASLD (Table S2); 25 SNPs were employed as IVs to study the causal relationship between PUFA and MASLD (Table S3).

### MR analysis

In this study, the inverse variance weighted (IVW), weighted median, weighted mode, simple mode and MR Egger methods were used to investigate the causal relationship between SFA, MUFA, PUFA and the outcome MASLD. The IVW method assumes all genetic variants are valid IVs, it employs a meta-analysis approach to combine the Wald ratios of the causal effects of each SNP, offering the most accurate estimates [31, 32]. The weighted median method yields a reliable estimate of the causal effect when at least 50% of the weight in the analysis comes from valid IVs [33]. The MR-Egger method permits for some degree of pleiotropic effect of IVs. It provides a causal effect via the slope coefficient of Egger regression, but it may decrease statistical power [23, 34]. The simple mode method is an unweighted mode of the empirical density function of causal estimates, while the weighted mode method is weighted by the inverse variance [35]. The weighted mode method is consistent even in the presence of invalid IVs when the largest number of similar individual instrument estimates comes from valid IVs [36]. The IVW method was used as the primary analysis method because of its high statistical power [37]. If the *P-*value of Cochran’s Q test was less than 0.05, the results of the MR analysis were analyzed using the IVW random effects model; otherwise, the fixed effects model was used. The weighted median, weighted mode, simple mode and MR‒Egger methods were used as supplementary methods. MR analysis was performed using the R package TwoSampleMR (version 0.5.8) in the R program (version 4.3.1).

### Sensitivity analysis

Our study employed an innovative approach, Radial MR analysis, using modified second-order weights to investigate potential outliers in MR analysis. This was implemented using the “RadialMR” package (version 1.1) in the R program (version 4.3.1), allowing the identification of outliers that could distort causal estimates and subsequent reanalysis after their exclusion [38]. The modified second-order weights computed by Radial MR take into account the first and second moments of the error term. This contrasts with traditional MR‒Egger regression, which considers only the first moment. By considering both moments, Radial MR can detect potential outliers that may bias MR estimates and remove them, providing more robust and reliable estimates of causal effects [39].

Sensitivity analysis was performed to determine the robustness of the MR results. Cochran’s Q test was used to detect heterogeneity in the data, with a *P-*value of less than 0.05 indicating the presence of heterogeneity; the MR-Pleiotropy RESidual Sum and Outlier (PRESSO) test was used to detect horizontal pleiotropy and identify outliers. If outliers were found, they were removed, and outlier-corrected MR analysis was performed to obtain an unbiased causal estimate. Directional pleiotropy was assessed via the MR‒Egger intercept test. Leave-one-out analysis was used to assess whether the MR results were strongly driven by specific SNPs. MR Steiger directionality test was used to further investigate the direction of causality between SFA, MUFA, PUFA and MASLD. Steiger directionality test was performed using the R package of Directionality_test in the R program. MR-PRESSO was conducted using the R package MR-PRESSO (version 1.0) in the R program (version 4.3.1).

### Assessment of severity of steatosis and hepatic lipid levels

Severity of hepatic steatosis was assessed based on hematoxylin-eosin (HE) staining. First, liver samples were fixed in a 5% formaldehyde solution for at least 48 h. Liver sections of 3 μm thickness were prepared. Sections were deparaffinized using xylol, rehydrated with descending grades of ethanol, and washed with distilled water. The sections were then stained with hematoxylin at room temperature for 15 min. Afterward, the sections were washed and kept in tap water for 15 min. Eosin was applied to the sections for 2 min at room temperature. Then, the slides were dehydrated, mounted, and covered with coverslips.

The stained sections were digitized using a whole slide scanner (L11600, Hamamatsu, Japan) equipped with NDP.view2 Plus Image viewing software (version U12388-02) at 40x magnification.

Two independent observers (F.X. and M.A.) assessed steatosis in the individual liver lobes of mice according to the “MASLD activity score (NAS)” criteria [40]. In case of discrepancies, the results were discussed with a board-certified pathologist (O.D.) to reach an agreement.

Hepatic triglyceride (TG) concentrations were determined using a colorimetric method according to the instruction of the TG quantitative assay kit (ab65336, Abcam). Lipids were extracted from 50 mg of snap-frozen liver tissue by homogenizing the samples in 0.5 ml of 5% NP40 (85124, Thermo Fisher Scientific) in double-distilled water using the SpeedMill Plus (Analytik Jena, Germany). The samples were gradually heated in a water bath at 95 °C for 5 min. The samples were cooled and subsequently reheated to ensure complete solubilization of all triglycerides in the solution. Centrifugation was conducted to remove any insoluble materials, and the resulting supernatants were diluted at a ratio of 1:20 with double-distilled water. All reactions were executed in duplicate to ensure reliability. The reactions were incubated for 20 min at room temperature with continuous agitation. After that, the triglyceride reaction mix was added to all reaction wells, followed by incubation for 60 min in the dark at room temperature. The output was measured using a microplate reader set to OD570.

### Statistical analysis

Animal experimental data analysis was performed with the statistical software SPSS 25.0 and SigmaPlot 13.0 (Statcon, Witzenhausen, Germany). The differences between groups were assessed using the one-way ANOVA test with LSD post hoc analysis (Data follow normal distribution or approximately normal distribution and homogeneity of variance) or Kruskal-Wallis test (Data does not follow normal distribution or homogeneity of variance). Statistical differences were considered significant when *P <* 0.05.

## Results

### SFA had no significant causal effect on MASLD risk

**Fig. 3:**
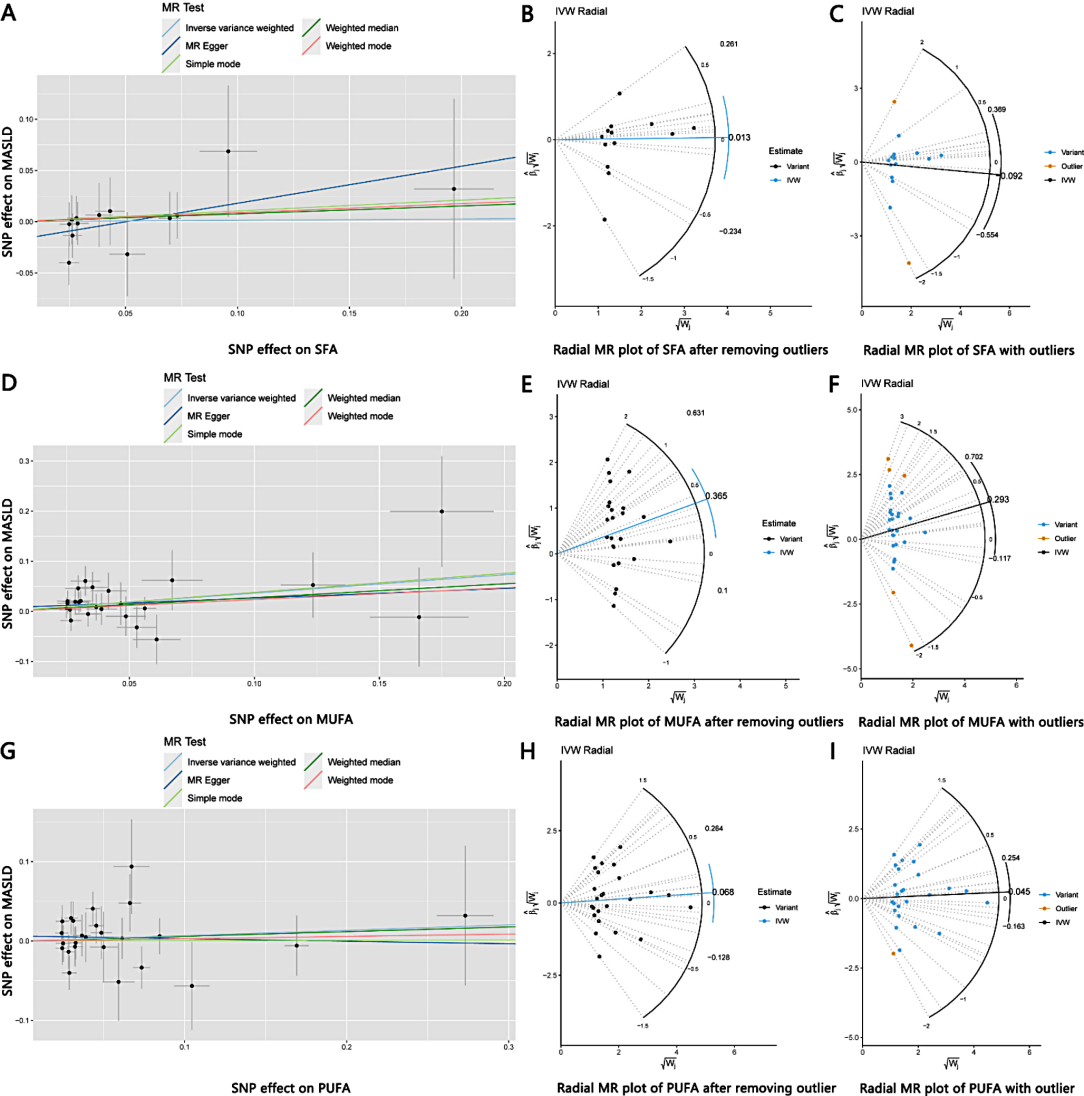
(**A**) Scatter plot demonstrating the causal relationship of SFA on MASLD; (**B**) Radial MR plot of SFA without outliers; (**C**) Radial MR plot of SFA with outliers; (**D**) Scatter plot demonstrating the causal relationship of MUFA on MASLD; (**E**) Radial MR plot of MUFA without outliers; (**F**) Radial MR plot of MUFA with outliers; (**G**) Scatter plot demonstrating the causal relationship of PUFA on MASLD; (**H**) Radial MR plot of PUFA without outlier; (**I**) Radial MR plot of PUFA with outlier

According to results of the IVW analysis, we found no significant causal effect of SFA on MASLD incidence (odds ratio, OR: 1.013, 95% CI: 0.739–1.388, *P =* 0.935). The results of the weighted median (OR: 1.080, 95% CI: 0.722–1.614, *P =* 0.708), weighted mode (OR: 1.092, 95% CI: 0.707–1.687, *P =* 0.698), simple mode (OR: 1.111, 95% CI: 0.636–1.940, *P =* 0.717) and MR Egger (OR: 1.436, 95% CI: 0.769–2.683, *P =* 0.281) analyses were also similar to the IVW analysis, the results did not reach statistical significance as well. The estimated effect sizes of the SNPs on both SFA and MASLD were displayed in scatter plots (Fig. 3A).

**Fig. 4:**
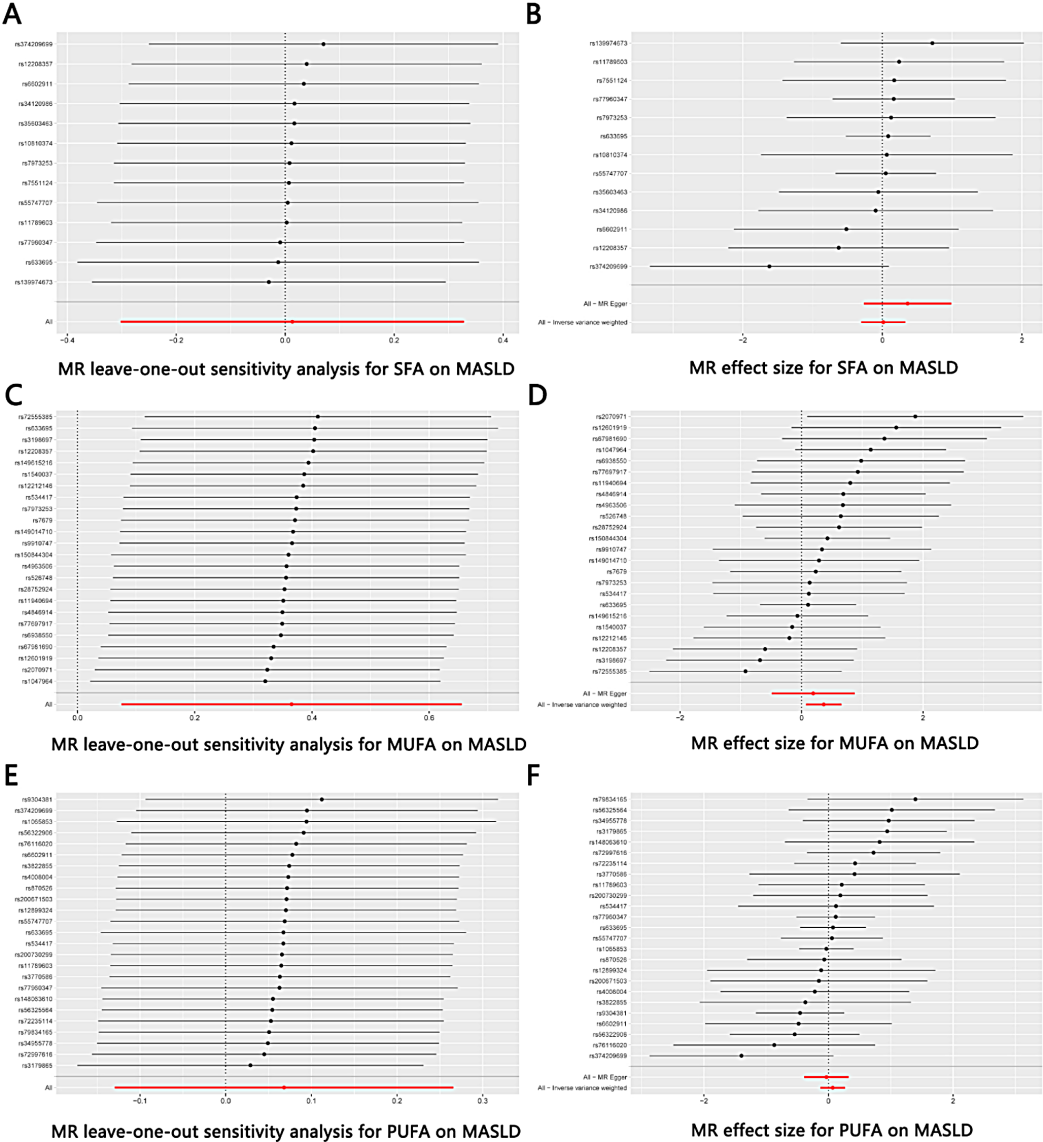
(**A**) Leave-one-out plot of SNPs associated with SFA and MASLD; (**B**) Forest plot of SNPs associated with SFA and MASLD; (**C**) Leave-one-out plot of SNPs associated with MUFA and MASLD; (**D**) Forest plot of SNPs associated with MUFA and MASLD; (**E**) Leave-one-out plot of SNPs associated with PUFA and MASLD; (**F**) Forest plot of SNPs associated with PUFA and MASLD

We used Cochrane’s Q test to detect heterogeneity in the data. The results showed no significant heterogeneity (*P* > 0.05). Besides, the result of the directional pleiotropy test by the Egger-intercept method was *P =* 0.232 > 0.05, indicating that the IVs did not significantly affect the outcome through pathways other than exposure. We used MR-PRESSO to confirm the absence of horizontal pleiotropy and outliers in the data (*P =* 0.942 > 0.05) (Table 2). Furthermore, the effect of SNPs on the incidence of MASLD was relatively stable in the leave-one-out analysis (Fig. 4A). The forest plot showed that SFA had no significant causal effect on MASLD risk (Fig. 4B).

**Fig. 5:**
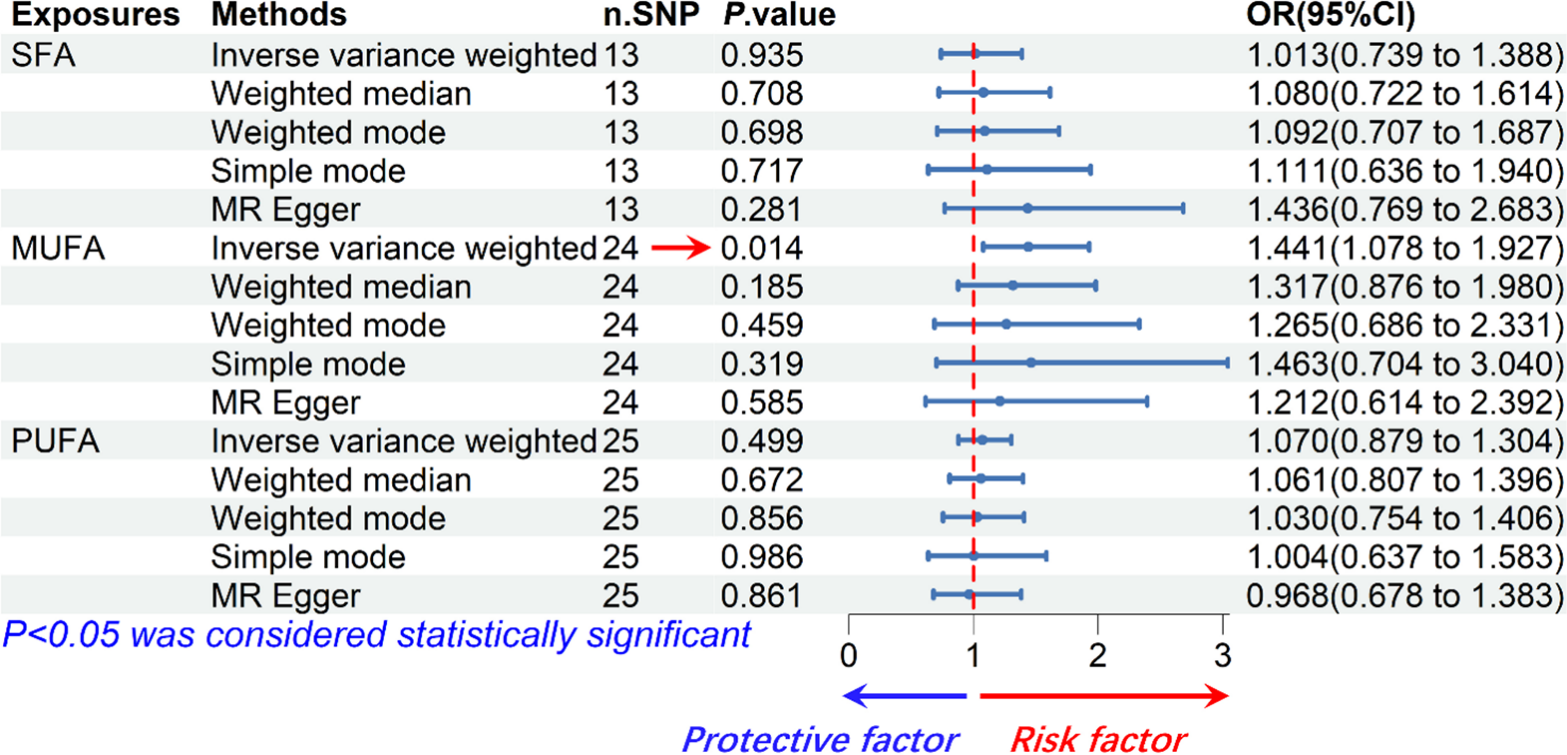
Forest plot for the causal relationship of SFA, MUFA and PUFA on MASLD. Overall, MUFA is a risk factor for the incidence of MASLD; SFA and PUFA have no significant causal effect on the incidence of MASLD

The Radial MR method is an advanced outlier detection technique that provides an additional layer of reliability for ensuring the robustness of research conclusion [38]. 2 outliers were detected among the IVs included in the analysis, and those outliers were removed. The overall shape and pattern of the plot suggests a possible weak link between SFA and MASLD (Fig. 3A, B, C). In summary, our findings demonstrated that SFA has no significant causal effect on MASLD (Fig. 5).

**Table 2:** Heterogeneity and pleiotropy analyses between SFA, MUFA, PUFA and MASLD

Exposures	Outcome	Cochrane’s Q				MR-PRESSO		Egger Intercept	
		IVW	*P*-val	MR Egger	*P*-val	Outliers	*P*-val	Intercept	*P*-val
SFA	MASLD	5.997	0.916	4.395	0.957	0	0.942	-0.018	0.232
MUFA	MASLD	17.240	0.797	16.936	0.767	0	0.826	0.007	0.587
PUFA	MASLD	21.332	0.619	20.892	0.588	0	0.600	0.006	0.514

Abbreviation: val, value

### MUFA had a positive causal effect on MASLD risk

According to the IVW analysis, MUFA were significantly correlated with MASLD (OR: 1.441, 95% CI: 1.078–1.927, *P =* 0.014). The results of the weighted median (OR: 1.317, 95% CI: 0.876–1.980, *P =* 0.185), weighted mode (OR: 1.265, 95% CI: 0.686–2.331, *P =* 0.459), simple mode (OR: 1.463, 95% CI: 0.704–3.040, *P =* 0.319) and MR Egger (OR: 1.212, 95% CI: 0.614–2.392, *P =* 0.585) analyses were similar to the IVW analysis, but the results did not reach statistical significance. The estimated effect sizes of the SNPs on both MUFA and MASLD were displayed in scatter plots (Fig. 3D).

There was no heterogeneity in the data according to the results of Cochrane’s Q test (*P* > 0.05). In addition, the data passed the directional pleiotropy test (*P =* 0.587 > 0.05). The results of MR-PRESSO showed no horizontal pleiotropy or outliers in the data (*P =* 0.826 > 0.05) (Table 2). Furthermore, no single SNP substantially violated the generalized effect of MUFA on MASLD incidence according to the leave-one-out analysis (Fig. 4C). The forest plot showed that MUFA may increase the risk of MASLD (Fig. 4D).

5 outliers were detected among the IVs via Radial MR analysis, and the outliers were removed. The overall shape and pattern of the plot supports the consistent association between MUFA and MASLD (Fig. 3D, E, F). In summary, our results revealed that MUFA was positively associated with the risk of MASLD, suggesting that MUFA might indeed be risk factor for the development of MASLD (Fig. 5). 

**Table 3:** MR Steiger directionality test for evaluating causal direction

Exposure	Outcome	snp_r2.exposure	snp_r2.outcome	Causal direction	Steiger P-value
SFA	MASLD	8.386×10^-^^03^	1.944×10^-^^04^	True	2.421×10^-^^118^
MUFA	MASLD	1.004×10^-^^02^	7.591×10^-^^04^	True	3.893×10^-^^104^
PUFA	MASLD	2.205×10^-^^02^	7.079×10^-^^04^	True	4.915×10^-^^292^

Furthermore, we used the MR Steiger directionality test to assess the direction of causality between SFA, MUFA, PUFA and MASLD, the results further confirmed that MUFA is a risk factor for MASLD (Table 3).

### PUFA had no significant causal effect on MASLD risk

According to the IVW analysis, PUFA was not significantly correlated with MASLD (OR: 1.070, 95% CI: 0.879–1.304, *P =* 0.499). The results of the weighted median (OR: 1.061, 95% CI: 0.807–1.396, *P =* 0.672), weighted mode (OR: 1.030, 95% CI: 0.754–1.406, *P =* 0.856) and simple mode (OR: 1.004, 95% CI: 0.637–1.583, *P =* 0.986) analyses were similar to the IVW analysis, the results did not reach statistical significance. In contrast, the result of the MR Egger (OR: 0.968, 95% CI: 0.678–1.383, *P =* 0.861) analysis differed from the above analysis, but the result did not reach statistical significance as well. The estimated effect sizes of the SNPs on both PUFA and MASLD were displayed in scatter plots (Fig. 3G).

There was no heterogeneity in the data according to the results of Cochrane’s Q test (*P* > 0.05). Besides, the data passed the directional pleiotropy test (*P =* 0.514 > 0.05). The results of MR-PRESSO showed no horizontal pleiotropy or outliers in the data (*P =* 0.600 > 0.05) (Table 2). Furthermore, no single SNP substantially violated the generalized effect of PUFA on MASLD incidence according to the leave-one-out analysis (Fig. 4E). The forest plot showed that PUFA had no significant causal effect on MASLD risk (Fig. 4F).

1 outlier was detected among the IVs via Radial MR analysis, and the outlier was removed. The overall shape and pattern of the plot suggests a possible weak link between PUFA and MASLD (Fig. 3G, H, I). In summary, our findings demonstrated that PUFA has no significant causal effect on MASLD (Fig. 5).

### High MUFA diet was associated with the development of hepatic lipid accumulation in mice

We collected the left lateral lobe (LLL), right median lobe (RML), right inferior lobe (RIL), and caudal lobe (CL) of the liver of mice for HE staining to assess the severity of hepatic steatosis. Three independent hepatologists, including a board-certified hepatopathologist, evaluated hepatic steatosis based on the staining results.

**Fig. 6:**
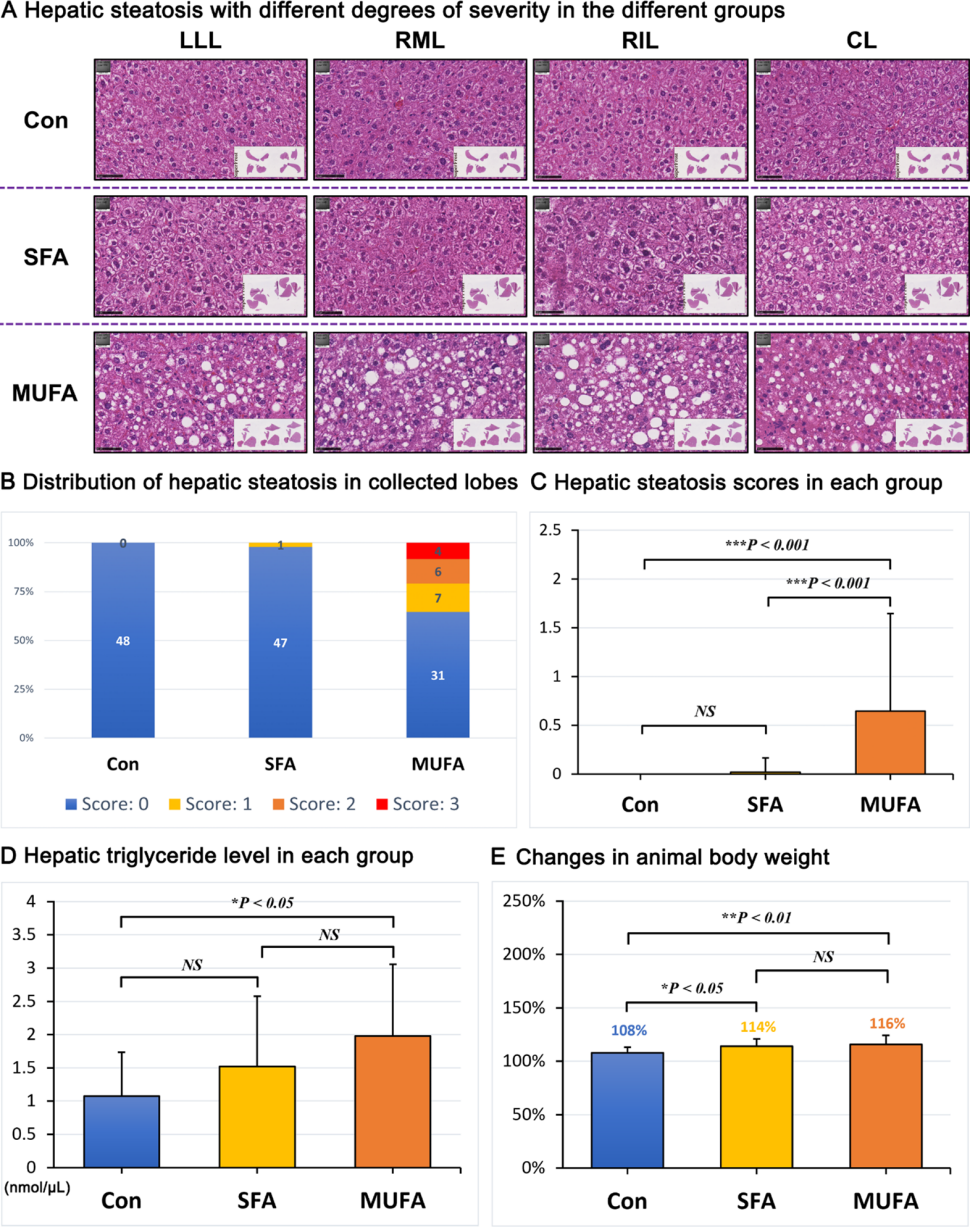
Assessment of hepatic steatosis in the three different groups of mice after 8 weeks of different dietary interventions. (**A**) HE staining showing different degrees of hepatic steatosis in different groups; (**B**) Distribution of semiquantitative grading of hepatic steatosis per liver lobe in the different groups of mice; (**C**) Hepatic steatosis scores in the different groups of mice; (**D**) Hepatic triglyceride levels in the different groups of mice; (**E**) Changes in body weight of mice in each group. Abbreviations: Con, normal control diet; SFA, saturated fatty acids diet “palmitic acid”; MUFA, monounsaturated fatty acids diet “oleic acid”; LLL, left lateral lobe; RML, right median lobe; RIL, right inferior lobe; CL, caudal lobe

**Fig. 7:**
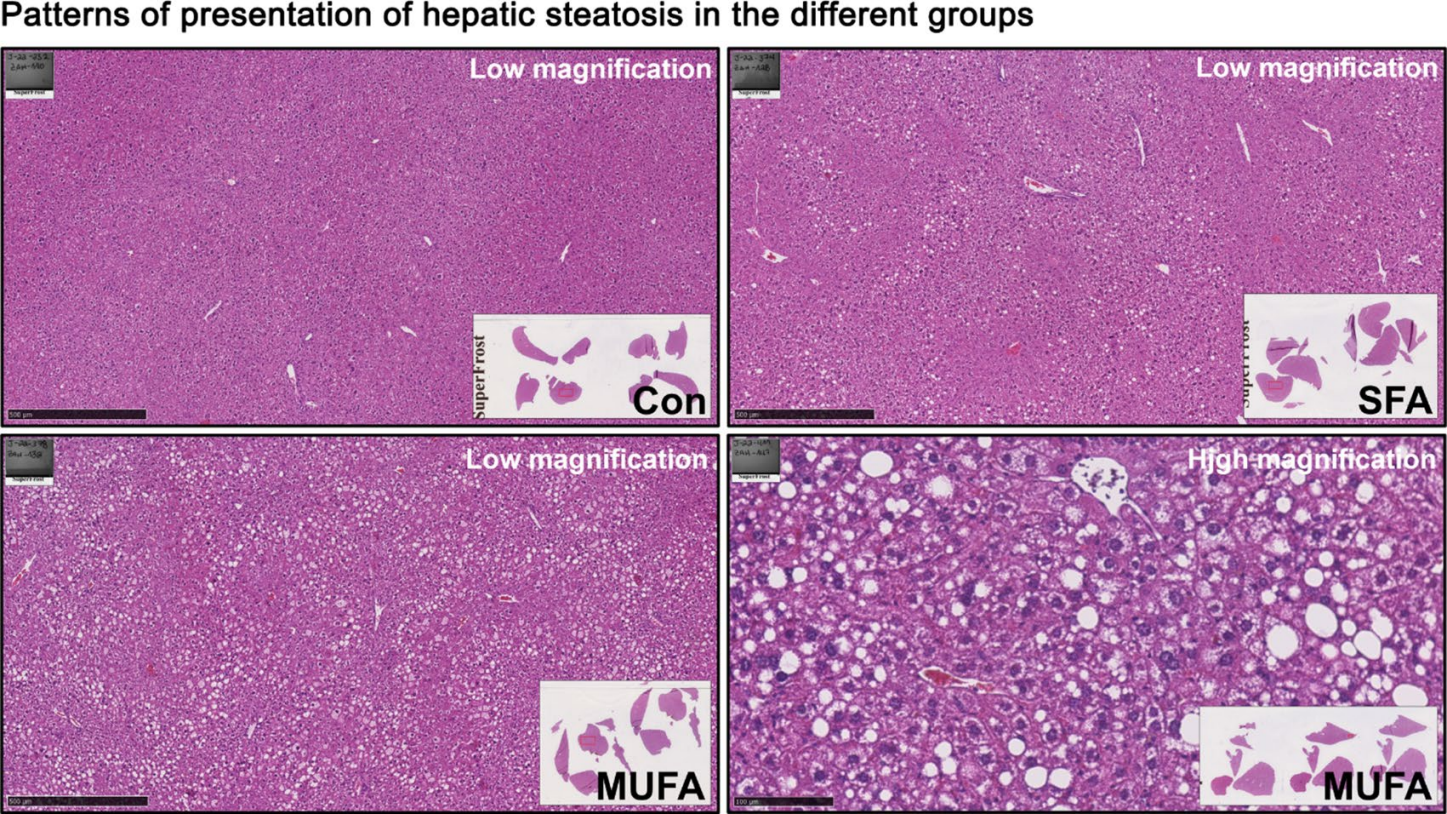
Hepatic steatosis type. HE staining showed periportal to midzonal mixed microvesicular and macrovesicular steatosis with different degrees of severity in the different groups. Abbreviations: Con, normal control diet; SFA, saturated fatty acids diet “palmitic acid”; MUFA, monounsaturated fatty acids diet “oleic acid”

We observed that 17 liver lobes of mice (17/48, 35.4%) fed with the MUFA-enriched (containing 18.6% HO sunflower oil) diet developed mild (7/17) to moderate (6/17) or severe (4/17) hepatic steatosis. (Fig. 6A, B). HE staining showed mixed microvesicular and macrovesicular steatosis in the periportal to midzonal area of the liver lobes (Fig. 7). The remaining liver lobes of the mice (31/48) in the group were morphologically normal and were almost free of fat-accumulating hepatocytes.

Interestingly, only 1 liver lobe of the mice (1/48, 2.1%) consuming the SFA-enriched diet (containing 9.3% palm oil and 9.3% palmitate ethyl ester) developed mild hepatic steatosis (1/1), whereas the other liver lobes of mice (47/48) in the group showed no signs of hepatic steatosis. Unsurprisingly, none of the animals consuming the control diet developed hepatic steatosis (Fig. 6A, B).

We statistically analyzed the hepatic steatosis scores of all four liver lobes in the three groups of animals. A significant difference was detected between the MUFA-enriched diet and the SFA-enriched diet and control diet (*P <* 0.001). In contrast, there was no significant difference between the SFA-enriched and the control diet (Fig. 6C).

We further examined hepatic triglyceride levels in mice by performing a colorimetric assay. The results of triglyceride assays showed that the MUFA-enriched diet significantly elevated hepatic triglyceride levels compared to the control diet (*P <* 0.05). Furthermore, the SFA-enriched diet seemed to also elevate triglyceride levels, but the differences did not reach statistical significance (Fig. 6D).

Moreover, we analyzed the changes in body weight before and after the experiment (weight at end divided by weight at start) in each group of animals. The results showed that the relative increase in body weight in mice from the MUFA-enriched diet group (16%) and the SFA-enriched diet group (14%) was significantly higher than that of the control group (*P* < 0.01 for MUFA and *P* < 0.05 for SFA). Furthermore, the relative increase in body weight in the MUFA-enriched diet group was slightly higher than that in the SFA-enriched diet group, but the difference was not statistically significant (Fig. 6E).

Overall, our animal results suggested that MUFA-enriched diet is more likely to cause MASLD than the SFA-enriched diet, which is consistent with the results of our TSMR study.

## Discussion

MASLD is a major global health threat due to its increasing prevalence. Currently, there are no registered drugs for the specific treatment of MASLD, and the primary clinical measures for the treatment of MASLD include a healthy diet and physical activity [41]. A healthy diet plays a pivotal role in preventing and improving MASLD [42], especially for individuals with limited mobility.

Fatty acids are essential nutrients in the diet and play a key role in maintaining normal physiological functions and cell structure [43]. Moreover, fatty acid intake appears to be associated with the development of MASLD. However, the effect of different types of fatty acids on the development of MASLD remains unclear (Table 4).

Dietary sources of fatty acids are mainly plant and animal products. In the daily human diet, common sources of SFA include palm oil, lard and butter, while common sources of UFA include sunflower oil, olive oil and fish oil. Previous studies have shown that SFA are commonly associated with adverse health effects. In contrast, UFA, particularly MUFA and n-3 PUFA, have been suggested to have protective effects against chronic diseases such as CVD and diabetes [18, 21, 43, 44].

In the last decade, these views have been challenged. Accumulating evidence suggests that the roles of SFA and UFA in diseases are controversially discussed [12, 13]. For example, a clinical study from the United States including 458 study participants showed that replacing dietary SFA with omega-6 linoleic acid (a PUFA) increased the rates of death from all causes, CHD and CVD [12]. Omega-6 PUFA, particularly arachidonic acid (AA), are highly abundant in cell membranes involved in inflammation. Omega-6-enriched dietary intake has been associated with inflammation, mainly because AA is a precursor of pro-inflammatory lipid mediators [45]. Dietary linoleic acid substantially upregulates cyclooxygenase-2, which converts AA to proinflammatory eicosanoids [46]. This could be a potential reason for the increased mortality caused by linoleic acid intake. Another study from Canada showed no clear associations between higher SFA intake and all-cause mortality, CHD, CVD, type 2 diabetes and ischemic stroke. However, the intake of trans-UFAs was associated with a 34% increase in all-cause mortality, a 28% increase in CHD mortality, and a 21% increase in CHD risk [13].

The liver serves as the central hub for the metabolism of fatty acids in the body, processing both SFA and UFA to meet the organism’s energy and other biological activity requirements [14, 17]. However, the specific roles of SFA, MUFA and PUFA in MASLD are uncertain. For example, Gentile et al. fed high saturated fat (HSAT) and high polyunsaturated fat (HPUF) diets to male Wistar rats for 4 and 24 weeks to study the effects of HSAT and HPUF diets on hepatic steatosis and liver injury. Their results showed that the sum of saturated fatty acids in triglycerides was substantially increased in the HSAT group compared to the HPUF group, while alanine aminotransferase, aspartate aminotransferase and hepatic caspase-3 activity levels were substantially upregulated as well [47]. Their results reflect that SFA seems to be more likely to cause hepatic lipid accumulation and liver injury compared to PUFA. Moreover, Liu et al. determined cellular lipid accumulation by Oil Red O staining after exposing HepG2 cells to PA or OA for 48 h. Their results showed that PA induced more lipid accumulation in HepG2 cells compared to OA [48]. The above results suggest that SFA appear to be more likely to cause hepatic steatosis than UFA. Contrary to these findings, the study by Eynaudi et al. showed that OA is more likely to induce hepatic steatosis than PA. Eynaudi et al. determined the cellular lipid content after exposing HepG2 cells to PA or OA for 24 h. The results of the study showed that OA treatment caused more and larger lipid droplets than PA treatment in HepG2 cells [14]. In addition, Ricchi et al. reported that OA treatment induced more pronounced triglyceride accumulation than PA treatment in HepG2, HuH-7 and WRL-68 cells. Interestingly, the level of PA-induced apoptosis was significantly greater than that of OA in the above three cell lines [15]. Moreover, we have shown previously that PA induces a pro-inflammatory response via activating Tumor necrosis factor-alpha (TNF-α) and increasing osteoclastogenesis. On the other hand, excess OA inhibits a pro-inflammatory response by fostering neutral triglyceride formation and protecting bone microarchitecture [49]. Furthermore, our recent study demonstrated that OA substitution abrogated PA-induced inflammatory signaling [50]. Overall, these results suggest that MUFA is more likely to induce hepatic steatosis compared to SFA, however, SFA is more likely to cause hepatic injury.

Clarifying the role of SFA, MUFA and PUFA in MASLD is clinically important for the prevention and mitigation of MASLD. MR refers to an analytical method for assessing the causal relationship between modifiable exposures and clinically relevant outcomes [51]. Nowadays, MR as an emerging causal research method in epidemiology for identifying risk factors, is a powerful complementary tool to enrich conventional observational studies [52, 53], as shown by our results.

Our study is the first MR analysis to explore the role of SFA, MUFA and PUFA in the incidence of MASLD. In this TSMR analysis, we comprehensively explored the relationship between SFA, MUFA, PUFA and MASLD. By analyzing a large amount of sample data, our results revealed that MUFA is indeed a risk factor for MASLD (OR: 1.441, *P =* 0.014), whereas SFA (OR: 1.013, *P =* 0.935) and PUFA (OR: 1.070, *P =* 0.499) do not appear to have a significant causal relationship with MASLD. Taken together, our TSMR study revealed a strong relationship between MUFA and MASLD.

These MR results are in line with the results of our animal experiments, which is based on a completely different approach. We examined the presence and severity level of hepatic lipid accumulation via tissue staining and colorimetric triglyceride assay. Our results showed that the MUFA diet significantly induced hepatic steatosis compared to the normal and SFA diet group of mice (*P <* 0.001). Moreover, the mild hepatic steatosis induced by the SFA diet did not reach statistical significance compared to that in the normal diet group. Overall, our findings suggest that excessive intake of MUFA is more likely to promote the development of MASLD.

**Table 4:** Role of SFA, MUFA and PUFA on liver lipid accumulation as shown in literature study

Year	Author	Research model	Intervention	Fatty acid rich type	Liver lipid accumulation
2001	Takeuchi et al. [54]	6 weeks old male Wistar rats	SFA; MUFA; PUFA	Tripalmitin; Olive oil; Linseed oil	MUFA-enriched diet caused more liver lipid accumulation compared to SFA and PUFA
2009	Ricchi et al. [15]	HepG2, HuH-7 and WRL-68 cells	SFA; MUFA	PA; OA	OA was more lipogenic but less apoptotic than PA in hepatocyte cultures
2014	Rosqvist et al. [55]	39 human participants	SFA; PUFA	PA; Linoleic acid	SFA-enriched diet induced more lipid accumulation than PUFA
2016	Lu et al. [56]	577 human participants	PUFA	Omega-3	PUFA-enriched diet improved liver lipid accumulation
2017	Duwaerts et al. [57]	Adult male C57BL/6J and C3H/HeOuJ mice	SFA; MUFA	Starch-PA; Starch-Oleate	MUFA-enriched diet induced more lipid accumulation than SFA
2018	Yan et al. [58]	1424 human participants	PUFA	Omega-3	PUFA-enriched diet decreased liver lipid accumulation
2020	Lee et al. [59]	1366 human participants	PUFA	Omega-3	PUFA-enriched diet improved liver lipid accumulation
2020	Zeng et al. [22]	Male Sprague- Dawley rats	SFA; MUFA	Fat and cholesterol; Olive oil	SFA-enriched diet promoted lipid accumulation; MUFA-enriched diet improved lipid accumulation
2021	Eynaudi et al. [14]	HepG2 cells	SFA; MUFA	PA; OA	OA was more lipogenic but less toxic than PA in hepatocyte culture
2021	Zhang et al. [60]	9 weeks old male Sprague-Dawley rats	SFA; PUFA	Lard; Fish oil	SFA-enriched diet promoted lipid accumulation; PUFA supplementation alleviated high-saturated fat diet-induced hepatic steatosis, inflammation and fibrosis
2023	Guo et al. [61]	C57BL/6J mice	MUFA	Olive oil	MUFA-enriched diet aggravated alcohol-induced liver steatosis
2023	Šmíd et al. [62]	3 months old male C57BL/6 mice	PUFA	Omega-3	PUFA-enriched diet ameliorated hepatic steatosis induced by a high-fat methionine choline-deficient diet

We conducted a literature study on the role of SFA, MUFA and PUFA in liver lipid accumulation and the results are shown in Table 4. Our findings are consistent with the conclusions of most studies, but also differ in some aspects from some studies. This may be due to the following reasons: (a) the species of study subjects; (b) the dose, method of administration and duration of the fatty acid intervention; (c) the composition of the lipid content of the recipe, etc. For example, in our study, we used male C57BL/6 mice as the study subjects, and the duration of the intervention with different diets was 8 weeks, while Zeng et al. [22] chose male Sprague-Dawley rats as the study subjects, the duration of their intervention was up to 32 weeks, and the composition of fatty acid diets was different. The above factors may have led to some differences in the conclusions between studies.

Our study has several major strengths:

First, we explored the causal relationships between SFA, MUFA, PUFA and MASLD from a genetic perspective based on newly published (all datasets published in 2021 and 2022) large sample GWAS data (Table 1). This approach is less susceptible to confounding factors and reverse causality than previous observational studies, making the research findings more reliable.

Second, it is a TSMR analysis that lends itself to causal inference. We established strict selection criteria for IVs. Only SNPs that met the relevance, independence and exclusion-restriction assumptions of the MR analysis could be selected as IVs.

Third, our study employed an innovative statistical approach. We used Radial MR analysis, using modified second-order weights to investigate potential outliers in MR analysis. Outliers in genetic association researches can often result from various factors such as genetic heterogeneity, genotyping errors and population stratification. Through Radial MR analysis, which involves detecting and excluding potential outliers, the observed correlations are ensured not to be spurious products of extreme observations [38, 39].

Fourth, we used comprehensive analytic tests to detect IVs for heterogeneity and pleiotropy, and all IVs passed the heterogeneity and pleiotropy tests. Besides, we executed a series of powerful MR methods to analyze the causal relationships among SFA, MUFA, PUFA and MASLD. Furthermore, the conclusion drawn from the TSMR were also validated in our in vivo experiment.

However, our study has several limitations:

First, not all MR analysis method yielded valid causal relationships. However, most of the analysis methods yielded similar results. Since all IVs passed the heterogeneity and pleiotropy tests, we chose the results of the IVW method with the highest test efficacy as the primary reference result [37].

Second, the source population of the dataset was European, which limits the applicability of the results to non-European populations. More research is needed in the future to validate the applicability of the results to other populations and ethnicities.

Third, the specific mechanism by which MUFA induces MASLD remains unclear. There are several potential links between these two conditions that have attracted the attention of researchers. It has been reported that hepatic Peroxisome proliferator-activated receptor-gamma (PPAR-γ) overexpression leads to lipid accumulation through transcriptional activation of genes responsible for lipid uptake and storage [63]. Many studies have shown enhanced expression of PPAR-γ and increased expression of lipogenic genes in animal models of fatty liver [64, 65]. Sterol regulatory element-binding protein-1 (SREBP-1) is a key factor in the regulation of lipid synthesis [66, 67]. It has been found that the greater degree of steatosis observed in hepatocytes after exposure to OA is associated with the activation of PPAR-γ and SREBP-1 [15]. Peroxisome proliferator-activated receptor-alpha (PPAR-α) is a crucial factor in the regulation of fatty acid oxidation. Studies have shown that PPAR-α activators modulate obesity in rodents via increasing hepatic fatty acid oxidation and decreasing circulating triglyceride levels [68–70]. It was found that unlike OA, PA treatment caused a substantial increase in PPAR-α expression in hepatocytes [15]. Therefore, MUFA may cause MASLD by regulating the expression of PPAR-γ, SREBP-1 and PPAR-α. Further exploration of the role of MUFA-induced lipid synthesis and its effect on fatty acid oxidation in MASLD may help to elucidate the specific mechanism linking MUFA and MASLD.

**Fig. 8:**
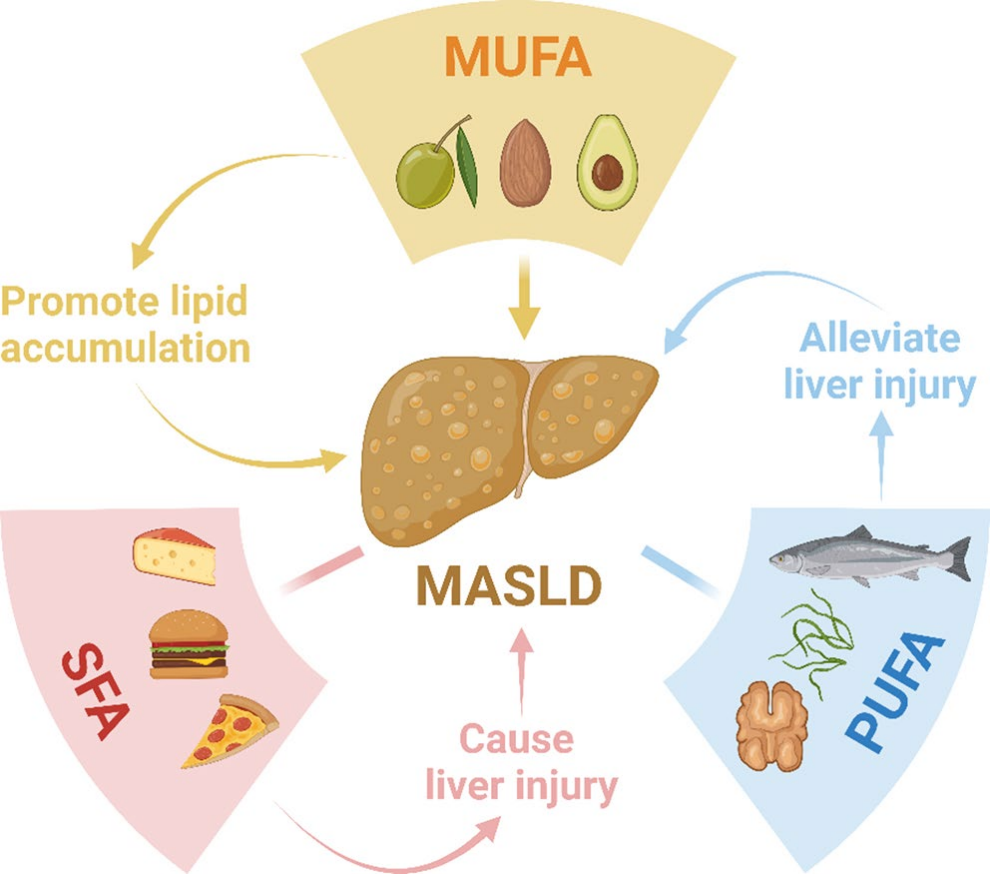
Effects of different fatty acids on the liver and MASLD. Abbreviations: MUFA, monounsaturated fatty acids; SFA, saturated fatty acids; PUFA, polyunsaturated fatty acids; MASLD, metabolic dysfunction-associated steatotic liver disease. We created the figure with “BioRender.com”

## Conclusion

In summary, our results support the idea of a causal relationship between MUFA and MASLD, but not between SFA or PUFA and MASLD. According to the TSMR and in vivo experiment results, a significant positive causal relationship between MUFA and MASLD was demonstrated (Fig. 8). Although the mechanism underlying the association between MUFA and MASLD is not fully understood, the results of the current MR analysis and animal experiment suggest that MUFA may play a crucial role in the pathogenesis of MASLD. Excessive intake of MUFA is more likely to cause MASLD. Therefore, replacing MUFA intake with a moderate intake of PUFA might help reduce the risk of MASLD.

## Electronic supplementary material

Below is the link to the electronic supplementary material.

Supplementary Material 1

## Data Availability

The SNP data that support the findings of this study are openly available in the OpenGWAS database (https://gwas.mrcieu.ac.uk/), reference number (SFA: ebi-a-GCST90092980; MUFA: ebi-a-GCST90092928, PUFA: ebi-a-GCST90092939 and MASLD: ebi-a-GCST90054782). The data generated from the MR study are available through the public database “FAIRDOMHub” (https://fairdomhub.org/documents/4343?version=1). Animal data are available from the corresponding author upon reasonable request.

## Abbreviations

AA: Arachidonic acid
CHD: Coronary heart disease
CI: Confidence interval
CL: Caudal lobe
CVD: Cardiovascular disease
GWAS: Genome-Wide Association Studies
HE: Hematoxylin-eosin
HPUF: High polyunsaturated fat
HSAT: High saturated fat
IV: Instrumental variable
IVW: Inverse variance weighted
LD: Linkage disequilibrium
LLL: Left lateral lobe
MASLD: Metabolic dysfunction-associated steatotic liver disease
MR: Mendelian randomization
MR-PRESSO: MR Pleiotropy RESidual Sum and Outlier
MUFA: Monounsaturated fatty acids
NAFLD: Non-alcoholic fatty liver disease
OA: Oleic acid
OR: Odds ratio
PA: Palmitic acid
PPAR-α: Peroxisome proliferator-activated receptor-alpha
PPAR-γ: Peroxisome proliferator-activated receptor-gamma
PUFA: Polyunsaturated fatty acids
RIL: Right inferior lobe
RML: Right median lobe
SFA: Saturated fatty acids
SREBP-1: Sterol regulatory element-binding protein-1
SNP: Single nucleotide polymorphism
TG: Triglyceride
TNF-α: Tumor necrosis factor-alpha
TSMR: Two-sample Mendelian randomization
UFA: Unsaturated fatty acids
