# Supplementary material for: The effects of saturated and unsaturated fatty acids on MASLD: a Mendelian randomization analysis and in vivo experiment
Source: Eur J Nutr. 2024 Dec 24;64(1):52. doi: 10.1007/s00394-024-03560-2 (PMC11668845; doi:10.1007/s00394-024-03560-2)
Supplement: Supplementary file 1 — Supplementary Material 1 [file 394_2024_3560_MOESM1_ESM.docx]

**The effects of saturated and unsaturated fatty acids on MASLD:** **A Mendelian randomization analysis and in vivo experiment**

**Fengming Xu ^1,2,3^, Mohamed Albadry ^1,4^, Annika Döding ^5^, Xinpei Chen ^1^, Olaf Dirsch ^6^, Ulrike Schulze-Späte ^5^ and Uta Dahmen ^1,*^**

^1^ Experimental Transplantation Surgery, Department of General, Visceral and Vascular Surgery, Jena University Hospital, Jena 07747, Germany;

^2^ Else Kröner Graduate School for Medical Students “JSAM”, Jena University Hospital, Jena 07747, Germany;

^3^ Department of Infectious Diseases, The First Affiliated Hospital of Zhejiang Chinese Medical University, Hangzhou 310006, China;

^4^ Department of Pathology, Faculty of Veterinary Medicine, Menoufia University, Shebin El Kom 6131567, Egypt;

^5^ Section of Geriodontics, Department of Conservative Dentistry and Periodontics, Jena University Hospital, Jena 07743, Germany;

^6^ Institute for Pathology, BG Klinikum Berlin, Berlin 12683, Germany.

* Correspondence: Uta.Dahmen@med.uni-jena.de; Tel.: +49-03641-9325350.

**Table S1** 13 genome-wide significant SNPs were used as IVs to investigate the causal relationship between SFA and MASLD

| **SNPs** | **CHR*** | **Position** | **EA*** | **OA*** | **Beta** | **EAF** | **Gene** | **SE*** | **F-Stat*** | ***P*-value** |
| --- | --- | --- | --- | --- | --- | --- | --- | --- | --- | --- |
| rs10810374 | 9 | 15306292 | C | A | 0.026 | 0.249 | TTC39B | 0.005 | 30.029 | 4.30×10^-08^ |
| rs11789603 | 9 | 107647019 | T | C | 0.043 | 0.109 | ABCA1 | 0.007 | 43.247 | 4.80×10^-11^ |
| rs12208357 | 6 | 160543148 | T | C | 0.051 | 0.070 | SLC22A1;  LOC124901452 | 0.008 | 40.319 | 2.20×10^-10^ |
| rs139974673 | 15 | 44027885 | C | T | 0.096 | 0.026 | CATSPER2P1 | 0.013 | 55.892 | 7.70×10^-14^ |
| rs34120986 | 8 | 11689763 | A | G | -0.025 | 0.372 | FDFT1 | 0.004 | 33.167 | 8.50×10^-09^ |
| rs35603463 | 6 | 32531745 | C | T | 0.029 | 0.567 | N/A | 0.005 | 34.412 | 4.50×10^-09^ |
| rs374209699 | 11 | 49104677 | GT | G | 0.025 | 0.358 | N/A | 0.005 | 30.170 | 4.00×10^-08^ |
| rs55747707 | 7 | 73037366 | A | G | -0.070 | 0.204 | MLXIPL | 0.005 | 189.397 | 4.30×10^-43^ |
| rs633695 | 15 | 58725839 | G | A | 0.073 | 0.292 | LIPC | 0.004 | 264.988 | 1.40×10^-59^ |
| rs6602911 | 13 | 114547372 | T | C | 0.026 | 0.360 | GAS6 | 0.004 | 38.452 | 5.60×10^-10^ |
| rs7551124 | 1 | 23785760 | T | C | 0.038 | 0.875 | ASAP3 | 0.006 | 38.737 | 4.80×10^-10^ |
| rs77960347 | 18 | 47109955 | G | A | 0.197 | 0.013 | LIPG | 0.018 | 122.325 | 2.00×10^-28^ |
| rs7973253 | 12 | 109878115 | G | A | 0.028 | 0.367 | MYO1H | 0.004 | 43.991 | 3.30×10^-11^ |

*Abbreviations: CHR, Chromosome; EA, Effect allele; OA, Other allele; SE, Standard error; F-Stat, F-statistics.

**Table S2** 24 genome-wide significant SNPs were used as IVs to investigate the causal relationship between MUFA and MASLD

| **SNPs** | **CHR** | **Position** | **EA** | **OA** | **Beta** | **EAF** | | **Gene** | **SE** | **F-Stat** | ***P*-value** |
| --- | --- | --- | --- | --- | --- | --- | --- | --- | --- | --- | --- |
| rs1047964 | 11 | 117156893 | T | G | -0.175 | | 0.010 | BACE1; RNF214 | 0.021 | 71.507 | 2.80×10^-17^ |
| rs11940694 | 4 | 39414993 | G | A | 0.025 | | 0.605 | KLB | 0.004 | 36.386 | 1.60×10^-09^ |
| rs12208357 | 6 | 160543148 | T | C | 0.053 | | 0.070 | SLC22A1;  LOC124901452 | 0.008 | 43.893 | 3.50×10^-11^ |
| rs12212146 | 6 | 161125454 | C | T | 0.049 | | 0.074 | PLG | 0.008 | 37.297 | 1.00×10^-09^ |
| rs12601919 | 17 | 65825374 | G | A | 0.030 | | 0.189 | BPTF | 0.005 | 32.001 | 1.50×10^-08^ |
| rs149014710 | 13 | 114756556 | T | C | -0.025 | | 0.431 | RASA3 | 0.005 | 30.303 | 3.70×10^-08^ |
| rs149615216 | 18 | 47106028 | T | C | 0.166 | | 0.011 | LIPG | 0.020 | 70.560 | 4.50×10^-17^ |
| rs150844304 | 15 | 43726625 | C | A | 0.123 | | 0.026 | TP53BP1 | 0.013 | 91.994 | 8.70×10^-22^ |
| rs1540037 | 18 | 47182664 | G | A | 0.033 | | 0.778 | LOC105372112 | 0.005 | 44.533 | 2.50×10^-11^ |
| rs2070971 | 7 | 44197583 | T | G | 0.032 | | 0.137 | GCK | 0.006 | 29.844 | 4.70×10^-08^ |
| rs28752924 | 6 | 31303922 | C | T | 0.030 | | 0.446 | N/A | 0.004 | 49.970 | 1.60×10^-12^ |
| rs3198697 | 16 | 15129940 | T | C | -0.027 | | 0.407 | PDXDC1 | 0.004 | 41.309 | 1.30×10^-10^ |
| rs4846914 | 1 | 230295691 | A | G | -0.031 | | 0.605 | GALNT2 | 0.004 | 53.786 | 2.20×10^-13^ |
| rs4963506 | 11 | 62187666 | A | G | 0.028 | | 0.196 | SCGB1A1;  LOC102723765 | 0.005 | 30.465 | 3.40×10^-08^ |
| rs526748 | 10 | 99772373 | T | G | 0.025 | | 0.554 | CRTAC1 | 0.004 | 38.113 | 6.70×10^-10^ |
| rs534417 | 1 | 23784965 | G | A | 0.039 | | 0.875 | ASAP3 | 0.006 | 40.114 | 2.40×10^-10^ |
| rs633695 | 15 | 58725839 | G | A | 0.056 | | 0.292 | LIPC | 0.004 | 156.329 | 7.20×10^-36^ |
| rs67981690 | 12 | 21343886 | G | A | 0.035 | | 0.129 | SLCO1B1 | 0.006 | 33.446 | 7.30×10^-09^ |
| rs6938550 | 6 | 20462138 | A | G | -0.042 | | 0.914 | E2F3 | 0.007 | 32.917 | 9.60×10^-09^ |
| rs72555385 | 7 | 73123473 | G | A | 0.061 | | 0.049 | STX1A | 0.009 | 41.588 | 1.10×10^-10^ |
| rs7679 | 20 | 44576502 | C | T | 0.037 | | 0.186 | PCIF1 | 0.005 | 49.195 | 2.30×10^-12^ |
| rs77697917 | 17 | 41840849 | T | C | 0.067 | | 0.029 | N/A | 0.012 | 30.404 | 3.50×10^-08^ |
| rs7973253 | 12 | 109878115 | G | A | 0.026 | | 0.367 | MYO1H | 0.004 | 38.395 | 5.80×10^-10^ |
| rs9910747 | 17 | 17433959 | C | A | 0.047 | | 0.062 | PEMT | 0.008 | 30.527 | 3.30×10^-08^ |

**Table S3** 25 genome-wide significant SNPs were used as IVs to investigate the causal relationship between PUFA and MASLD

| **SNPs** | **CHR** | **Position** | **EA** | **OA** | **Beta** | **EAF** | | **Gene** | **SE** | **F-Stat** | ***P*-value** |
| --- | --- | --- | --- | --- | --- | --- | --- | --- | --- | --- | --- |
| rs1065853 | 19 | 45413233 | T | G | -0.169 | | 0.081 | N/A | 0.007 | 523.615 | 6.90×10^-116^ |
| rs11789603 | 9 | 107647019 | T | C | 0.049 | | 0.109 | ABCA1 | 0.006 | 56.615 | 5.30×10^-14^ |
| rs12899324 | 15 | 58555709 | T | C | -0.025 | | 0.267 | N/A | 0.005 | 30.186 | 3.90×10^-08^ |
| rs148063610 | 10 | 45998984 | CAAATAAAT | C | 0.031 | | 0.237 | N/A | 0.005 | 41.739 | 1.00×10^-10^ |
| rs200671503 | 11 | 116817978 | TTA | T | -0.050 | | 0.058 | N/A | 0.009 | 33.634 | 6.60×10^-09^ |
| rs200730299 | 1 | 55491853 | C | A | -0.037 | | 0.194 | N/A | 0.005 | 46.035 | 1.20×10^-11^ |
| rs3179865 | 6 | 31324194 | A | G | 0.043 | | 0.398 | HLA-B;  MIR6891 | 0.004 | 95.543 | 1.40×10^-22^ |
| rs34955778 | 16 | 15139594 | C | T | -0.030 | | 0.420 | PDXDC1;  NTAN1 | 0.004 | 53.810 | 2.20×10^-13^ |
| rs374209699 | 11 | 49104677 | GT | G | 0.029 | | 0.358 | N/A | 0.004 | 41.855 | 9.80×10^-11^ |
| rs3770586 | 2 | 169828995 | T | C | -0.024 | | 0.484 | ABCB11 | 0.004 | 35.483 | 2.60×10^-09^ |
| rs3822855 | 6 | 116316882 | T | G | 0.024 | | 0.401 | FRK | 0.004 | 35.479 | 2.60×10^-09^ |
| rs4008004 | 9 | 15300968 | A | C | 0.032 | | 0.222 | TTC39B | 0.005 | 44.330 | 2.80×10^-11^ |
| rs534417 | 1 | 23784965 | G | A | 0.039 | | 0.875 | ASAP3 | 0.006 | 41.444 | 1.20×10^-10^ |
| rs55747707 | 7 | 73037366 | A | G | -0.061 | | 0.204 | MLXIPL | 0.005 | 150.986 | 1.10×10^-34^ |
| rs56322906 | 19 | 11346155 | A | G | -0.104 | | 0.035 | DOCK6 | 0.011 | 91.888 | 9.20×10^-22^ |
| rs56325564 | 17 | 45766771 | A | G | 0.024 | | 0.483 | N/A | 0.004 | 36.587 | 1.50×10^-09^ |
| rs633695 | 15 | 58725839 | G | A | 0.085 | | 0.292 | LIPC | 0.004 | 365.151 | 2.10×10^-81^ |
| rs6602911 | 13 | 114547372 | T | C | 0.028 | | 0.360 | GAS6 | 0.004 | 45.801 | 1.30×10^-11^ |
| rs72235114 | 19 | 45444524 | CT | C | 0.045 | | 0.295 | N/A | 0.005 | 95.014 | 1.90×10^-22^ |
| rs72997616 | 11 | 75474195 | A | C | -0.066 | | 0.094 | DGAT2-DT | 0.007 | 91.960 | 8.80×10^-22^ |
| rs76116020 | 16 | 69385641 | G | A | -0.059 | | 0.044 | TMED6 | 0.010 | 36.283 | 1.70×10^-09^ |
| rs77960347 | 18 | 47109955 | G | A | 0.273 | | 0.013 | LIPG | 0.018 | 243.345 | 7.30×10^-55^ |
| rs79834165 | 6 | 34889423 | C | T | -0.067 | | 0.034 | ANKS1A | 0.011 | 36.840 | 1.30×10^-09^ |
| rs870526 | 2 | 20369562 | T | C | -0.033 | | 0.521 | N/A | 0.004 | 66.355 | 3.80×10^-16^ |
| rs9304381 | 18 | 47158234 | T | C | 0.073 | | 0.818 | LOC105372112 | 0.005 | 198.722 | 4.00×10^-45^ |
